# Combinations of Linear Type Traits Affecting the Longevity in Hungarian Holstein-Friesian Cows

**DOI:** 10.3390/ani11113065

**Published:** 2021-10-27

**Authors:** Evelin Török, István Komlósi, Viktor Szőnyi, Béla Béri, Gábor Mészáros, János Posta

**Affiliations:** 1 Department of Animal Husbandry, Institute of Animal Science, Biotechnology and Natural Conservation, Faculty of Agricultural and Food Sciences and Environmental Management, University of Debrecen, H-4032 Debrecen, Hungary; torok.evelin@agr.unideb.hu (E.T.); komlosi@agr.unideb.hu (I.K.); beri@agr.unideb.hu (B.B.); postaj@agr.unideb.hu (J.P.); 2 Doctoral School of Animal Science, University of Debrecen, H-4032 Debrecen, Hungary; 3 Sz-Consult Ltd., H-4080 Hajdúnánás, Hungary; szonyiv.75@gmail.com; 4 Department of Sustainable Agricultural Systems, Division of Livestock Sciences, University of Natural Resources and Life Sciences, 1180 Vienna, Austria

**Keywords:** Holstein-Friesian cattle, the combination of linear type traits, survival analysis

## Abstract

**Simple Summary:**

The selection for high-level milk production shortened the productive life of cows. In Hungary, the average number of lactations is only ca. 2.1. At the same time, the longevity, health status, milk production and reproduction were affected by the linear type traits, confirmed by several research studies. These studies, however, looked at the association of the linear type traits with longevity, using only one trait at a time. To take into account the connections between linear type traits, their combinations based on statistical analyses were considered. Identification of the risk ratio of various trait combinations could support corrective mating and bull selection.

**Abstract:**

Several research studies confirm the association of the linear type traits with longevity, but only with one trait at a time. The aim of our study was to analyse the influence of combinations for linear type traits on longevity in the Hungarian Holstein-Friesian cows. Data were provided by four herds; the filtered dataset consisted of 17,717 cows. From the 14 available linear type traits, the most important combinations were identified based on principal components and cluster analysis. From the six identified combinations, only three (chest width-body depth, fore udder attachment-udder depth, angularity-rear udder height) proved to have a significant effect on longevity. A wide chest and deep body caused a high-risk ratio of culling. The lowest risk ratio was observed with cows having intermediate chest width and intermediate body depth. Very angular cows having low rear udder height were at the highest risk of culling. The lowest culling risk was found in cows with a lack of angularity and high rear udder height. Weak and loose fore udder along with deep udder had increased culling risk. Strong and tight fore udder subclasses were the most favourable as their risk ratios decreased towards the shallowing of udder depth.

## 1. Introduction

Longevity can be described in various ways and measures; it covers productivity of life when defined as the time period between first calving and culling [1]. Longevity is an important trait in the selection of dairy cattle, as it contributes to profitability. Longer productive life increases the profits and decreases the heifer replacement costs [2], as well as decreasing the methane emission per kg of milk produced [3], thus reducing the environmental footprint of the milk industry [4]. According to [5], the production life was less than 3–4.5 years, whereas the natural lifespan was approximately 20 years to [6]. The average number of lactations ranges from 2.1 to 2.2 in the Hungarian Holstein-Friesian population. To improve this low number, longevity is taken into account with the weight of 10% within the selection index of the Hungarian population. In many developed countries, longevity was within the selection index, but in many developing countries it has not been included in the performance index [1]. It has been shown that linear type traits indirectly affect the longevity of cattle. The linear type traits are connected with the body size, the rump, the feet and legs and the udder system. Several authors estimated a strong relationship between the udder traits and longevity. In Dadpasaand et al. [7], it was reported a close connection between a productive life and fore udder attachment, udder depth as well as udder cleft, respectively. In Imbayarwo-Chikosi et al. [8], it was reported that fore teat placement, udder depth, fore udder attachment, and rear teat placement had a higher impact on longevity compared with other linear type traits. According to [9,10,11], the strong fore udder attachment increased longevity. In Morek-Kopec and Zarnecki [12], it was estimated a higher risk ratio for animals having deep udder, weak and loose fore udder attachment, narrow udder width or low rear udder height. The higher rear udder height, the shallow udder as well as the strong fore udder attachment increased the longevity reported by [13].

In Tsurata et al. [9], it was reported that cows having straighter legs and steeper foot angles had a longer productive life. According to [12], the extremely sickled legs and low foot angle increased the culling. In Imbayarwo-Chikosi et al. [8], a lower risk ratio for extremely low and extremely steep foot angles was computed. In Tsurata et al. [9], a positive connection between smaller body size and longer productive life was found. In Vacek et al. [14], it was revealed the cows with smaller body depth and chest width had a longer productive life. In contrast, Sewalem et al. [15] found that the lowest risk ratio was estimated for intermediate body depth; a shallow or deeper body and narrow chest width increased the culling risk. Narrow chest width and the short rump height also increases the risk ratio according to [8]. In Morek-Kopec and Zarnecki [12], it was reported that the highest risk of culling was found for more angular and narrower or wide rump cows. In Zavadilová et al. [16] as well as Zavadilová and Štipková [17], it was reported that by increasing the score of angularity, the productive life decreased.

Previous studies mostly examined single linear type traits and their relation with longevity, but the relationship between the combinations of linear type traits and longevity have not been studied yet. Several analyses carried out the analysis of the relationship among conformation traits using principal component analysis (PCA), a method which determines the relative contribution of factors to the variation of the traits belonging to each identified factor. In Chu and Shi [18], four important factors were found for Beijing Holstein cows using PCA. These factors were: the relationship within the body and rump traits (body strength, body depth, rump length, rump width); the udder traits (fore udder attachment, rear udder width, udder cleft, teat placement); factor determined by the dairy form, rear leg side view, rear udder height, and udder depth; and the final factor affected by the rump and foot angle. In Kern et al. [19], two factors were extracted using maximum likelihood method: the first was dependent upon udder texture, udder cleft, loin strength, bone quality, final score, rear udder height and width, whereas the second was affected by stature, top line, chest width, body depth, fore udder attachment, angularity and final score. In Ali et al. [20], five factors were reported using the PCA method for Canadian Holstein cows. Factor I. was in relation with the mammary system, these factors were affected by udder texture, median suspensory, fore udder attachment, fore teat placement, rear attachment height, and rear attachment width. Factor II. was determined by stature, body size, chest width, and pin width, whereas Factor III. was dependent upon bone quality. Factor IV. was related to the feet and legs traits (foot angle, set of the rear leg); finally, Factor V. was affected by the loin strength and pin setting. In Pundir et al. [21], three factors for were observed for Kankrej cows, also using PCA. The first factor described the cow body (body length, heart girth, paunch girth, height at knee, height at wither), the second presented the front view/face of the cow (ear length, face length, face width, horn length, neck length) and the third factor was determined by the back of the cow.

The increase in or at least maintenance of longevity is of great importance in Holstein-Friesian cattle worldwide. Several research studies have confirmed the association of the conformation traits with longevity, but only with one trait at a time. This approach could not estimate the interaction effect of traits. In our study, combinations of the traits based on statistical analyses were considered. This approach might give information about the joint effects of linear type traits. The aim of our study was to analyse the influence of these combinations for linear type traits on longevity in the Hungarian Holstein-Friesian cows.

## 2. Materials and Methods

The dataset consisted of 17,717 Holstein-Friesian cows, from 4 representative commercial herds. The cows born after 2000 and first calved from 2002 to 2019 were used in the analysis. The data of the cows were gathered by the Hungarian Holstein Association and by the herds. The pedigree file used during survival analysis contained 26,122 cattle. 

Longevity was defined as the time from first calving to culling or censoring. The measurement variable was the number of lactations, as mentioned in [22]. The data of cows alive at the time of data collection were censored, with a total of 22.3% right-censored records. Major culling reasons are described in Table S1.

Linear type traits were evaluated following [23] and were judged once on first-parity cows within the interval 30 days after first calving and before the end of lactation. The combinations of linear type traits were created based on principal component analysis and cluster analysis. 

**Figure 1:**
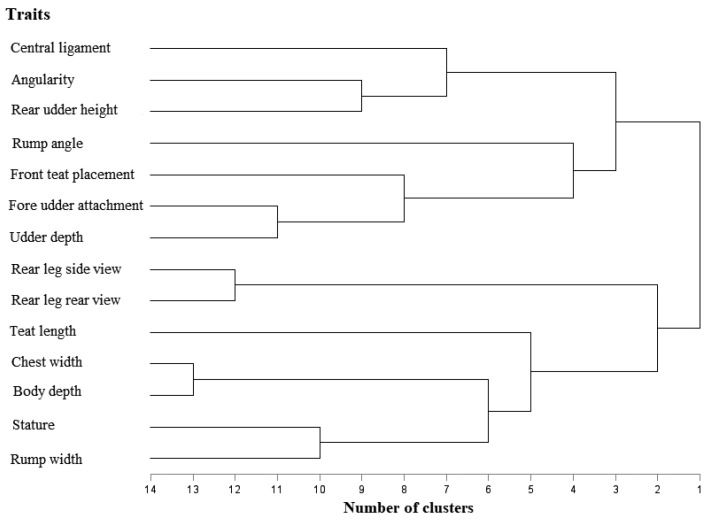
Dendrogram of linear type traits.

The principal component analysis was carried out using SAS PROC FACTOR [24] software package with method = principal and rotate = varimax options. The principal component analysis was used to define the contribution of factors to the total variation of the traits. To maximize the variance of the squared loadings of factors, the Varimax rotation of H. F. Kaiser [25] was used. The rotation method takes into consideration the correlation among background variables. In this case, background variables are not independent of one another. Factors having eigenvalues higher than 1.0 were taken into account during further work. Traits were supposed to be related to each factor in the case of correlation coefficient ≥ |0.5|. Two trait principal components (Table 1) and two trait clusters (Figure 1) were used as combinations during the analysis. These combinations were related to the body capacity (Factor I., Factor IV.), to the mammary system and udder structure (Factor III. and Factor V.) and the structure of the rear legs (Factor II.). 

The cluster analysis was carried out and presented using SAS PROC VARCLUS and TREE [24] software packages. Figure 1 shows the separation of linear type traits by cluster analysis. The highest relationship was observed between chest width and body depth, rear leg side view and rear leg rear view, fore udder attachment and udder depth, stature and rump width as well as between angularity and rear udder height.

Based on the results of principal component analysis (a) and cluster analysis (b) following combination of linear type traits were examined:

1. Chest width–body depth (a, b)
2. Rear legs rear view–rear legs side view (a, b)
3. Fore udder attachment–udder depth (a, b)
4. Stature–rump width (a, b)
5. Central ligament–front teat placement (a)
6. Angularity–rear udder height (b)

Scores of linear type traits are on a 1–9 scale, which were further joined to three groups: 1–3 denoted as 1, 4–6 denoted as 2 and 7–9 denoted as 3.

For further analyses, the effect of the defined combinations of linear type traits on longevity were studied. The number of lactations as the discrete measurement of time was chosen as the dependent variable. The model contained the fixed effect of the herd (1, 2, 3, 4), the year of birth (2000–2017), the age at first calving (≤22 months, 23 months, 24 months, 25 months, 26 months, 27 months, ≥28 months) and combinations of linear type traits and the random effect of the animal. The year of calving was a time-dependent covariate changing every year. The structure of the model was:

(1) $$\lambda \left(t\right)=\lambda_{0}\left(t\right)exp\left\{\sum_{i}f_{i}\left(t\right)+c_{j}+cwbd_{k}+rwsw_{l}+uaud_{m}+srw_{n}+cltp_{o}+aruh_{p}\right\}$$

where $\sum_{i}f_{i}\left(t\right)$ is the sum of fixed environmental effects (herd, birth year, age at first calving), $c_{j}$ is the time dependent covariate year of calving, $cwbd_{k}$ is the combination of chest width-body depth, $rwsw_{l}$ is the combination of rear legs rear view-rear legs side view, $uaud_{m}$ is the combination of fore udder attachment-udder depth, $srw_{n}$ is the combination of stature-rump width, $cltp_{o}$ is the combination of central ligament-front teat placement,$\;aruh_{p}$ is the combination of angularity-rear udder height.

Relationships among different factors (herd, age at first calving, age at calving, combinations of linear type traits) and longevity were estimated using the Weibull model in the Survival Kit program [26]. The risk ratios showed the relative risk of culling, compared with the reference class (where the risk ratio = 1). In our study, culling was the death or slaughter of cows.

The heritability of longevity based on the animal model and was computed using the following formula:

(2) $$h^{2}=\frac{\sigma_{g}^{2}}{\frac{1}{p}+\sigma_{g}^{2}}$$

$h^{2}=$ heritability.
$\sigma_{g}^{2}=$ genetic variance estimated using the Survival Kit.
p = proportion of uncensored records [27].

## 3. Results

The significances of all effects are shown in Table 2. Animal, age at first calving, year of calving, chest width and body depth, fore udder attachment and udder depth as well as angularity and rear udder height were significant effects on longevity.

**Figure 2:**
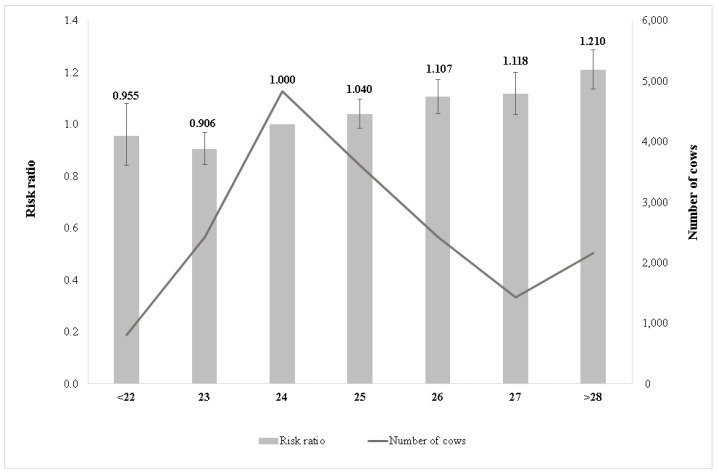
Effect of age at first calving on the relative culling risk.

The final model contained only significant effects/combinations, non-significant effects/combinations were removed from the model and not used during further analysis. Figure 2 presents the risk ratios of the effect of age at first calving on longevity. The highest risk of culling was estimated for cows first calved after 28 months. Cows having first calving after 28 months had a 21% higher risk of culling compared with the reference class (age at first calving: 24 months, risk ratio = 1.0). The lowest risk ratio was observed in cows having their first calving at 23 months, with about 9% less risk of culling compared with the reference class.

**Figure 3:**
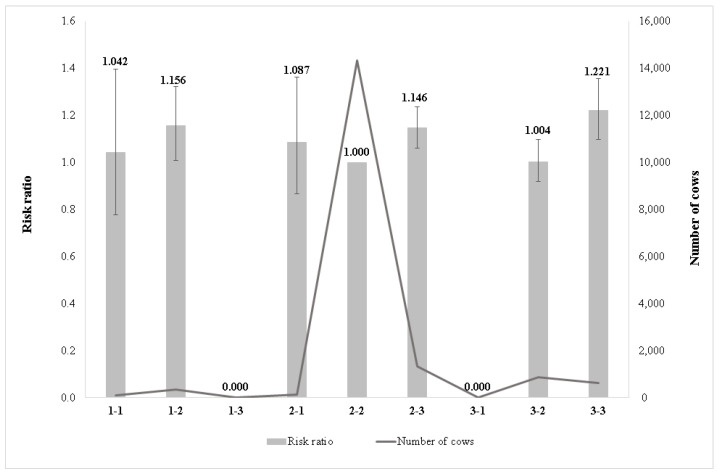
Effect of combination of chest width and body depth on the relative culling risk. **1-1**: narrow chest–shallow body depth; **1-2**: narrow chest–intermediate body depth; **1-3**: narrow chest–deep body; **2-1**: intermediate chest width–shallow body depth; **2-2**: intermediate chest width–intermediate body depth; **2-3**: intermediate chest width–deep body; **3-1**: wide chest–shallow body depth; **3-2**: wide chest–intermediate body depth; **3-3**: wide chest–deep body.

Figure 3 shows the effect of the combination of chest width and body depth on the relative culling risk. The lowest risk ratio was observed in class 2-2 (intermediate chest width and intermediate body depth). In this case, the cows have optimal body weight, and this combination is gentler on the joints as well as having a more favourable effect on the foot structure. The stall floor made from concrete is part of the typical housing system in Hungarian farms; under such environmental conditions, the intermediate chest width and body depth are advantageous. Cows having a wide chest and deep body (class 3-3) had the highest culling risk compared with the reference group (class 2-2). This could be related to higher body weight, as a heavier body puts larger pressure on the foot which is unfavourable, mainly on the concrete floor. It was followed by cows with a narrow chest and intermediate body depth (class 1-2) and an intermediate chest width and deep body (class 2-3). The tendency of risk ratios was similar for narrow chest (1-1, 1-2) and wide chest (3-2, 3-3) subclasses as risk ratios had increased along with body depth deepening, whereas for intermediate chest width subclasses the lowest culling ratio was estimated for intermediate body depth. There were no observations for categories 1-3 and 3-1. Overall, the risk ratio was higher in the case of deeper bodies.

**Figure 4:**
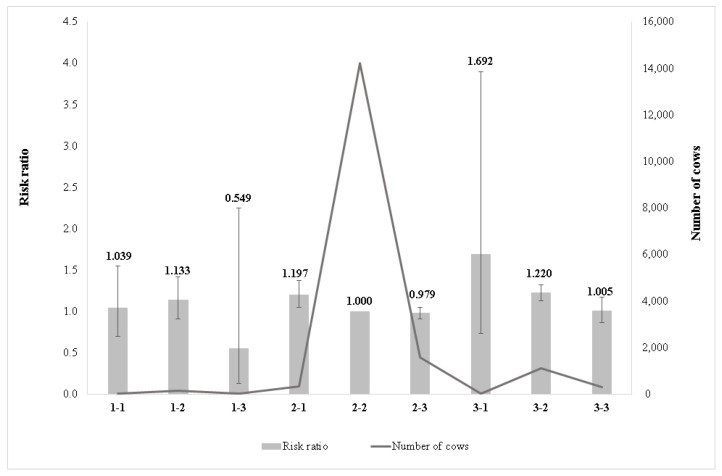
Effect of combination of angularity and rear udder height on the relative culling risk. **1-1**: lacks angularity–low rear udder height; **1-2**: lacks angularity–intermediate rear udder height; **1-3**: lacks angularity–high rear udder height; **2-1**: intermediate angularity–low rear udder height; **2-2**: intermediate angularity–intermediate rear udder height; **2-3**: intermediate angularity–high rear udder height; **3-1**: very angular–low rear udder height; **3-2**: very angular–intermediate rear udder height; **3-3**: very angular–high rear udder height.

Figure 4 shows the risk ratios of the effect of the combination of angularity and rear udder height on longevity. The highest risk ratio was found for the very angular cows with low rear udder height (class 3-1). The second highest risk ratio was observed in class 3-2 (very angular with intermediate rear udder height) and the third in class 2-1 (intermediate angularity with low rear udder height). The lowest culling risk was found in cows with a lack of angularity and high rear udder height (class 1-3). The high-yielding Holstein-Friesian cows usually have high rear udder height. The cows having low rear udder height, generally have a lower milk yield, which might increase the risk of culling.

**Figure 5:**
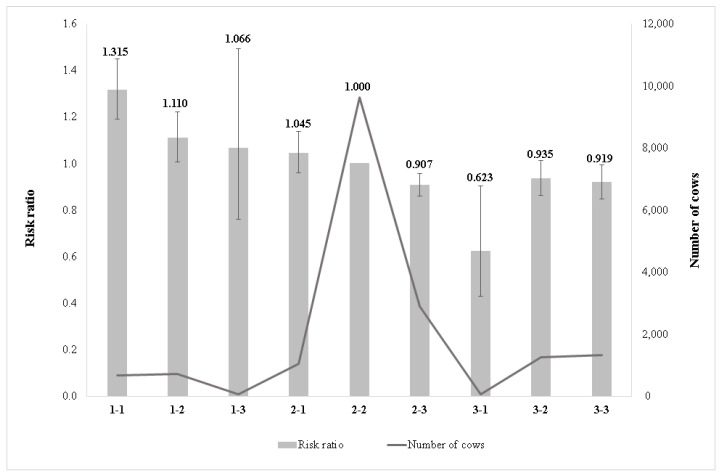
Effect of combination of fore udder attachment and udder depth on the relative culling risk. **1-1**: weak and loose fore udder–deep udder; **1-2**: weak and loose fore udder–intermediate udder depth; **1-3**: weak and loose fore udder–shallow udder depth; **2-1**: intermediate fore udder–deep udder; **2-2**: intermediate fore udder–intermediate udder depth; **2-3**: intermediate fore udder–shallow udder depth; **3-1**: strong and tight fore udder–deep udder; **3-2**: strong and tight fore udder–intermediate udder depth; **3-3**: strong and tight fore udder–shallow udder depth.

The relationship between the combination of fore udder attachment and udder depth and culling risk was shown in Figure 5. The highest risk ratio was estimated for class 1-1. Cows having weak, loose, and deep fore udders had a higher risk of culling than what was estimated for the reference group (class 2-2). The tendency of risk ratios was similar for weak and loose fore udder (1-1, 1-2, 1-3) and intermediate fore udder (2-1, 2-2, 2-3) subclasses as risk ratios had decreased towards shallower udder depth. Strong and tight fore udder subclasses were the most favourable, and the smallest risk ratio was estimated for class 3-1, though the number of cows was low for this class.

After adjusting for the fix effects, the animal variance was estimated to be 0.169, which resulted in heritability h2 = 0.12 of longevity, taking into account the 22.3% censoring rate (*p* = 0.777 was the proportion of uncensored records).

## 4. Discussion

Analyses of the combinations of linear type traits, the chest width-body depth, the fore udder attachment-udder depth and the angularity-rear udder height had a significant effect on longevity (Table 2). There is no information about the combination of linear type traits in the scientific literature as researchers evaluated the effect of traits separately. Influence of interaction between linear type traits might be revealed in this way. This might help breeders during their work with notification to problematic conformation combinations. Our estimations were in line with [8,28,29,30] as they recognized the relationship between longevity and udder depth. The connections between rear udder height and longevity [14,17] and between fore udder attachment and longevity [7,11,29], were confirmed also by other authors. Surprisingly, the combination related to leg and foot traits (rear legs rear view-rear legs side view) were not significant, contrary to other authors who reported leg and foot traits having the greatest impact on longevity [28,31,32].

The standard errors and significance of differences between classes within an effect were not commonly shown in previous studies using survival analysis. In this paper, however, we compute and show the 95% confidence intervals for the estimated risk ratios, in order to put the risk ratios and respective number of individuals for that class into perspective. We emphasize that only the significant effects were kept in the final model (see Table 2), but the pairwise differences between classes within an effect were not significantly different in all cases. Only significant differences are presented and discussed in detail from here on. From this perspective, it is perhaps better to refer to risk ratios between respective classes with non-significant differences as tendencies. As the standard errors were not published previously, we could compare our results with other studies based only on estimated risk ratios.

The later age at first calving increased the culling risk (Figure 2). Cows first calved at 23 months of age had significantly smaller risk ratios than those of first calving at later ages. Our results were in agreement with [33] as they reported higher culling risk for age at first calving in later age groups. In M’hamdi et al. [34], a 1.43 relative culling risk for 39 months of first calving age was estimated. In Chirinos et al. [35], it was found that cows having age at first calving over 34 months had the higher relative culling risk for the Spanish Holstein-Friesian Andalusian sub-population. In Páchová et al. [36], a lower risk of culling for cows younger at first calving was reported for Holstein cattle. Our finding was similar to those reported by [37] as they found a positive relationship between 2.0–2.5 years of age at first calving and longevity index. According to [38], the later age at first calving leads to decreased reproductive performance and it might be the main reason for reduced longevity.

The class of intermediate chest width and intermediate body depth was the most favourable and was significantly different from the class of wide chest and deep body, which was the most unfavourable for longevity (Figure 3). Our results were in line with [17] as they reported that cows having a deep body and wide chest had a lower breeding value for longevity. Similarly, Buenger et al. [13] recognized that deep cows had a negative deviation of the length of productive life for dairy cows, whereas [10] connected the increased longevity with shallow body depth for a Holstein cattle population. In contrast, Vacek et al. [14] reported a longer productive life for cows with smaller body depth and chest width scoring for the Czech Holstein cow population. In Morek-Kopec and Zarnecki [12], it was estimated that the wider chest width increases the risk of culling for Polish Holstein-Friesian cattle. In Imbayarwo-Chikosi et al. [8], the highest risk of culling was found for cows with a very narrow chest in South African Holstein cattle, as a score of 1 for chest width showed the highest risk of culling (2.47). According to [11], taller and larger cows had a longer productive life in Holstein cows.

Class 3-2 significantly differed from class 2-2 and class 2-3. The very angular cows with low rear udder height seemed to have shorter longevity; in contrast, the lack of angularity and high rear udder height increased the longevity (Figure 4). In Setati et al. and Buenger et al. [10,13], a positive relationship was found between high rear udder height and longevity. Cows with a lack of angularity mainly have a better body condition, which could result in better energy supply for the animal. The same tendency was estimated for the entire data set, when the risk ratio decreased along with the increased score in rear udder height, except for class 1-2. Our result was similar to [16], where the most angular cows had poorer longevity for Czech Holstein cows. They reported that the angular cows were more sensitive to environmental conditions, which led to a shorter productive life. In Zavadilová and Štipková [17], it was found the negative genetic correlation between angularity and longevity (−0.31) which supports our result. Despite a shorter productive life, angular cows are supposed to have better milking performance, as was found by [39,40,41] for various Holstein populations.

The weak, loose and deep fore udder significantly differed from classes 2-1, 2-2, 2-3, 3-1, 3-2, 3-3, as it increased the culling, whereas the strong and tight fore udder was the most favourable on longevity (Figure 5). Our estimation was in agreement with [14], as they found a strong relationship between above-average fore udder attachment and productive life. Based on their results, cows having moderately deep udders appeared to have the longest productive life. In Morek-Kopec and Zarnecki [12], the highest risk of culling for cows with deep udder and weak and loose fore udder attachment was reported. According to [16], cows with deep udders had a higher risk of culling. In [7], it was reported that extremely deep or shallow udders increased the culling risk. According to their results, the increasing score of fore udder attachment decreased the culling risk in Holstein-Friesian cattle. In Schneider et al. [11], the relationship between strongly attached udder and productive life was reported for Holstein cows. In Caraviello et al. [30], a strong relationship was found between low scores for fore udder attachment and high culling risk. In Imbayarwo-Chikosi et al. [8], it was reported that the cows with fore udder attachment and udder depth scores of 6-8 had a lower risk of culling. In Setati et al. [10], it was concluded that the cows with strongly attached and shallow udders had longer herd lives. In Buenger et al. [13], it was recognized that the cows with shallow udders had a longer life. A strong and tight fore udder attachment decreased the risk ratio, and a weak and loose fore udder increased it, similarly to our findings. In Hungarian dairy farms, the main health problem and culling reason is mastitis. The somatic cell count (SCS) and mastitis were also in close relation [42]. The udder depth and fore udder attachment might be in a relationship with mastitis and the somatic cell count. According to [43], the deep udders and weak fore udder attachment showed the highest SCS. In Berry et al. [44], the connection was found between tighter fore udder attachment and SCS for primiparous dairy cows. In Nash et al. [45], it was reported that cows with shallower and strongly attached fore udders had lower clinical mastitis incidence.

Our estimation about the heritability of longevity (number of lactations) was quite similar to the results of [46], as 0.11 was reported as heritability. Besides the number of lactations, longevity could be evaluated using the number of days between the first calving and the culling. In Páchová et al. and Raguž et al. [36,47], lower heritability of longevity (0.041, 0.075) was found for Croatian Simmental cattle using this measurement value, whereas [34] and [48] reported higher values of heritability (0.19 and 0.18, respectively). Based on the review of [1], the heritability of longevity varied between 0.01–0.30 by using different models.

## 5. Conclusions

The estimation of the relationship between linear type trait combinations and longevity using survival analysis methodology was focused on in our study. Such a joint evaluation of linear type traits and their impact would be also beneficial, according to opinions of type trait evaluators and breeders in Hungary. From the 14 available type traits, the most important combinations were identified based on principal component and cluster analysis. From the six identified combinations, only three proved to have a significant effect on longevity in the Hungarian Holstein Friesian cattle. The most important type trait combinations for longevity were those describing the body size and the udders. A wide chest and deep body resulted in a higher culling risk. Very angular cows having low rear udder height were under the highest risk of culling among angularity–rear udder height combinations, whereas the lack of angularity and high rear udder height was the most favourable combination. Weak and loose fore udder along with deep udder had increased culling risk compared with intermediate cows. These findings have shown that longevity is genetically inherited and are expected to support corrective mating and help to select proper bulls in the Hungarian Holstein-Friesian population.

## Figures and Tables

**Table 1:** Eigenvalues and percentage of the total variance, factors and factor loadings after rotation based on analysis of linear type traits.

Traits	Factor I.	Factor II.	Factor III.	Factor IV.	Factor V.
Eigenvalues	1.618	1.553	1.537	1.397	1.257
Variance of eigenvalues %	11.6	11.1	10.9	9.9	8.9
Stature	0.19	−0.05	0.19	0.73	−0.04
Chest width	0.72	0.07	0.08	0.32	−0.02
Body depth	0.89	0.04	−0.08	0.15	0.03
Angularity	−0.06	−0.09	0.01	0.08	0.06
Rump angle	−0.04	−0.03	−0.07	0.04	−0.01
Rump width	0.16	0.07	−0.09	0.82	0.04
Rear leg rear view	0.01	−0.88	−0.03	0.05	0.00
Rear leg side view	0.09	0.86	0.09	0.09	0.00
Fore udder attachment	0.19	0.14	0.83	−0.08	0.01
Rear udder height	0.04	0.08	0.21	0.05	0.10
Central ligament	−0.12	−0.01	−0.04	0.07	0.83
Udder depth	−0.36	0.00	0.78	0.16	0.21
Front teat placement	0.19	0.01	0.35	−0.08	0.72
Teat length	0.10	0.04	0.04	0.02	0.03

**Table 2:** Significant effects of some factors affecting longevity.

Factors		*p*-Values
Random effect	Animal	<0.0001
Fix effects	Herd	0.4232
	Year of birth	0.1257
	Age at first calving	<0.0001
Time-dependent covariate	Year of calving	<0.0001
Combinations of linear type traits	Chest width–body depth	<0.0001
	Rear legs rear view–rear legs side view	0.8774
	Fore udder attachment–udder depth	<0.0001
	Stature–rump width	0.3721
	Central ligament–front teat placement	0.9188
	Angularity–rear udder height	<0.0001

## Data Availability

Not applicable.

## Supplementary Materials

The following are available online at https://www.mdpi.com/article/10.3390/ani11113065/s1, Table S1: Distribution of major culling causes (%).

## References

[1] Hu H., Mu T., Ma Y., Wang X.P., Ma Y. (2021). Analysis of Longevity Traits in Holstein Cattle: A Review. Front. Genet..

[2] Van Arendonk J.A.M. (1991). Use of profit equations to determine relative economic value of dairy cattle herd life and production from field data. J. Dairy Sci..

[3] Grandl F., Furger M., Kreuzer M., Zehetmeier M. (2019). Impact of longevity on greenhouse gas emissions and profitability of individual dairy cows analysed with different system boundaries. Animal.

[4] Dallago G.M., Wade K.M., Cue R.I., McClure J.T., Lacroix R., Pellerin D., Vasseur E. (2021). Keeping dairy cows for longer: A critical literature review on dairy cow longevity in high milk-producing countries. Animals.

[5] Kerslake J.I., Amer P.R., O’Neil P.L., Wong S.L., Roche J.R., Phyn C.V.C. (2018). Economic costs of recorded reasons for cow mortality and culling in a pasture-based dairy industry. J. Dairy Sci..

[6] Najafabadi H.A., Mahayari S.A., Edriss M.A., Strapakova E. (2016). Genetic analysis of productive life length in Holstein dairy cows using weibull proportional risk model. Arch. Anim. Breed..

[7] Dadpasaand M., Miraei-Ashtiani S.R., Shahrebabak M.M., Torshizi R.V. (2008). Impact of conformation traits on functional longevity of Holstein cattle of Iran assessed by a Weibull proportional hazards model. Livest. Sci..

[8] Imbayarwo-Chikosi V.E., Ducrocq V., Banga C.B., Halimani T.E., Van Wyk J.B., Maiwashe A., Dzama K. (2016). Impact of conformation traits on functional longevity in South African Holstein cattle. Anim. Prod. Sci..

[9] Tsurata S., Misztal I., Lawler T.J. (2005). Changing definition of productive life in US Holsteins: Effect on genetic correlation. J. Dairy Sci..

[10] Setati M.M., Norris D., Banga C.B., Benyi K. (2004). Relationship between longevity and linear type traits in Holstein cattle population of Southern Africa. Trop. Anim. Health Prod..

[11] Schneider M.P., Dürr J.W., Cue R.I., Monardes H.G. (2003). Impact of type traits on functional herd life in Quebec Holsteins assessed by survival analysis. J. Dairy Sci..

[12] Morek-Kopec M., Zarnecki A. (2012). Relationship between conformation traits and longevity in Polish Holstein Friesian cattle. Livest. Sci..

[13] Buenger A., Ducrocq V., Swalve H.H. (2001). Analysis of survival in dairy cows with supplementary data on type scores and housing systems from a region of Northwest Germany. J. Dairy Sci..

[14] Vacek M., Stípková M., Němcová E., Bouška J. (2006). Relationships between conformation traits and longevity of Holstein cows in the Czech Republic. Czech J. Anim. Sci..

[15] Sewalem A., Kistemaker G., Migilior F., Van Doormaal B.J. (2004). Analysis of the relationship between type traits and functional survival in Canadian Holsteins using a Weibull proportional hazards model. J. Dairy Sci..

[16] Zavadilová L., Němcová E., Štípková M. (2011). Effect of type traits on functional longevity of Czech Holstein cows estimated from a Cox proportional hazard model. J. Dairy Sci..

[17] Zavadilová L., Štipková M. (2012). Genetic correlations between longevity and conformation traits in the Czech Holstein population. Czech J. Anim. Sci..

[18] Chu M.X., Shi S.K. (2002). Phenotypic factor analysis for linear type traits in Beijing Holstein Cows. Asian-Australas. J. Anim. Sci..

[19] Kern E.L., Cobuci J.A., Costa C.N., McManus C.M., Pimentel C.M.M. (2014). Factor analysis of linear type traits and their relation with longevity in Brazilian Holstein cattle. Asian-Australas. J. Anim. Sci..

[20] Ali A.K., Koots K.R., Burnside E.B. (1998). Factor analysis of genetic evaluations for type traits of Canadian Holstein sires and cows. Asian-Australas J. Anim. Sci..

[21] Pundir R.K., Singh P.K., Singh K.P., Dangi P.S. (2011). Factor analysis of biometric traits of Kankrej cows to explain body conformation. Australas. J. Anim. Sci..

[22] Brotherstone S., Veerkamp R.F., Hill W.G. (1997). Genetic parameters for a simple predictor of the lifespan of Holstein-friesian dairy cattle and its relationship to production. Anim. Sci..

[23] ICAR (2018). Section-5—ICAR Guidelines for Conformation Recording of Dairy Cattle, Beef Cattle, Dual Purpose Cattle and Dairy Goats. https://www.icar.org/Guidelines/05-Conformation-Recording.pdf.

[24] SAS Institute Inc (2007). SAS Online Doc^®^ 9.2..

[25] Kaiser H.F. (1958). The varimax criterion for analytic rotation in factor analysis. Psychometrika.

[26] Mészáros G., Kadlečík O., Kasarda R., Sölkner J. (2013). Analysis of longevity in the Slovak Pinzgau population—Extension to the animal model. Czech J. Anim. Sci..

[27] Mészáros G., Sölkner J., Ducrocq V. (2013). The Survival Kit: Software to analyze survival data including possibly correlated random effects. Comput. Methods Programs Biomed..

[28] Larroque H., Ducrocq V. (2001). Relationships between type and longevity in the Holstein breed. Genet. Sel. Evol..

[29] Dube B., Dzama K., Banga C.B., Norris D. (2009). An analysis of the genetic relationship between udder health and udder conformation traits in South African Jersey cows. Animal.

[30] Caraviello D.Z., Weigel K.A., Gianola K.A. (2004). Analysis of the relationship between type traits and functional survival in US Holstein Cattle using a Weibull Proportional Hazards Model. J. Dairy Sci..

[31] Royal M.D., Pryce J.E. (2002). The genetic relationship between commencement of luteal activity and calving interval, body condition score production and linear type traits in Holstein-Friesian dairy cattle. J. Dairy Sci..

[32] Sawa A., Boqucki M., Krężel-Czopek S., Neja W. (2013). Relationship between conformation traits and lifetime production efficiency of cows. ISRN Vet. Sci..

[33] Zavadilová L., Štipková M. (2013). Effect of age at first calving on longevity and fertility traits for Holstein cattle. Czech J. Anim. Sci..

[34] M’hamdi N., Aloulou R., Bouallegue M., Brar S.K., Hamouda M.B. (2010). Study on functional longevity of Tunisian Holstein dairy cattle using a Weibull proportional hazard model. Livest. Sci..

[35] Chirinos Z., Carabano M.J., Hernández D. (2007). Genetic evaluation of length of productive life in the Spanish Holstein-Friesian population: Model validation and genetic estimation. Livest. Sci..

[36] Páchová E., Zavadilová L., Sölkner J. (2005). Genetic evaluation of the length of productive life in Holstein cattle in the Czech Republic. Czech J. Anim. Sci..

[37] Haworth G.M., Tranter W.P., Chuck J.N., Cheng Z., Wathes D.C. (2008). Relationships between age at first calving and first lactation milk yield, and lifetime productivity and longevity in dairy cows. Vet. Rec..

[38] Cielava L., Jonkus D., Paura L. (2017). The effect of cow reproductive traits on lifetime productivity and longevity. World Acad. Sci. Eng. Technol. Int. J. Anim. Vet. Sci..

[39] Campos R.V., Cobuci J.A., Kern E.L., Costa C.N., McManus C.M. (2015). Genetic parameters for linear type traits and milk, fat, and protein production in Holstein cows in Brazil. Asian-Australas J. Anim. Sci..

[40] Tapki K., Guzey Y.Z. (2013). Genetic and phenotypic correlations between linear type traits and milk production yields of Turkish Holstein Dairy Cows. Greener J. Agri. Sci..

[41] Battagin M., Sartori C., Biffani S., Penasa M., Cassandro M. (2013). Genetic parameters for body condition score, locomotion, angularity, and production traits in Italian Holstein cattle. J. Dairy Sci..

[42] Rogers G.W., Hargrove G.L., Lawlor T.J., Ebersole J.L. (1991). Correlations among linear type traits and somatic cell counts. J. Dairy Sci..

[43] Němcová E., Štípková M., Zavadilová L., Bouška J., Vacek M. (2007). The relationship between somatic cell count, milk production and six linearly scored type traits in Holstein cows. Czech J. Anim. Sci..

[44] Berry D.P., Buckley F., Dillon P., Evans R.D., Veerkamp R.F. (2004). Genetic relationships among linear type traits, milk yield, body weight, fertility and somatic cell count in primiparous dairy cows. Ir. J. Agr. Food Res..

[45] Nash D.L., Rogers G.W., Cooper J.B., Hargrove G.L., Keown J.F., Hansen L.B. (2000). Heritability of clinical mastitis incidence and relationships with sire transmitting abilities for somatic cell score, udder type traits, productive life, and protein yield. J. Dairy Sci..

[46] Imbayarwo-Chikosi V.E., Ducrocq V., Banga C.B., Halimani T.E., Van Wyk J.B., Maiwashe A., Dzama K. (2017). Estimation of genetic parameters for functional longevity in the South African Holstein cattle using a piecewise Weibull proportional hazards model. J. Anim. Breed. Genet..

[47] Raguž N., Jovanovac S., Mészáros G., Sölkner J. (2014). Linear vs. piecewise Weibull model for genetic evaluation of sires for longevity in Simmental cattle. Mljekarstvo.

[48] Vukasinovic N., Moll J., Casanova L. (2001). Implementation of a routine genetic evaluation for longevity based on survival analysis techniques in dairy cattle populations in Switzerland. J. Dairy Sci..

