# Supplementary material for: Combinations of Linear Type Traits Affecting the Longevity in Hungarian Holstein-Friesian Cows
Source: Animals (Basel). 2021 Oct 27;11(11):3065. doi: 10.3390/ani11113065 (PMC8614554; doi:10.3390/ani11113065)
Supplement: Supplementary file 1 [file animals-11-03065-s001.zip › animals-1335212-supplementary.pdf]

Table S1. Distribution of major culling causes (%)

Reason for culling	Cows (n)	Cows (%)
Reproduction problems	3 469	25.2
Metabolic disorders	2 701	19.6
Low milk yield	2 138	15.5
Udder problems	2 012	14.6
Others	1 894	13.8
Foot or leg problems	1 430	10.4
Unknown	123	0.7
Total	12 767	100

Table 1. presents the most important culling reasons. Reproduction problems (25.2%) were the major culling cause, followed by metabolic disorders (19.6%), low milk yield (15.5%), udder problems (14.6%), others (13.8%), foot or leg problems (10.4%) and finally unknown problems (0.7%). The ranking of reproduction problems was in agreement with [1], [2] and [3], as they reported the most important cause of culling was the poor fertility for the Holstein-Friesian dairy cows. Frequency of udder problems in our study was in align with [2] and was smaller compared to [3] findings. According to [4], the frequent culling causes were diseases (38.7%), reproduction problems (36.0%), udder disorders (7.7%), lameness (3.7%) and death (12.7%) for Holstein dairy cows.

References

1. Mohammadi, G.R.; Sedighi, A. Reasons for culling of Holstein dairy cows in Neishaboor area in northeastern Iran. *Iran J. Vet. Res*. **2009**, *10*, 278-282. https://doi.org/10.22099/IJVR.2009.1709

2. Adamczyk, K.; Makulska, J.; Jagusiak, W.; Węglarz, A. Associations between strain, herd size, age at first calving, culling reason and lifetime performance characteristics in Holstein-Friesian cows. *Animal* **2016**, *11.* 327-334. https://doi.org/10.1017/S1751731116001348

3. Chiumia, D.; Chagunda, M.G.G.; Macrae, A.I.; Roberts, D.J. Predisposing factors for involuntary culling in Holstein–Friesian dairy cows. *J. Dairy Res.* **2012,** *80,* 45–50. https://doi:10.1017/S002202991200060X

4. Boujenane, I. Reasons and risk factors for culling of Holstein dairy cows in Morocco. *J. Livest. Sci. Technol.* **2017**, *5*, 25-31. https://doi: 10.22103/jlst.2017.1661
